# Genetic and phenotypic diversity of *Fusarium oxysporum* f. sp. *niveum* populations from watermelon in the southeastern United States

**DOI:** 10.1371/journal.pone.0219821

**Published:** 2019-07-18

**Authors:** Aparna Petkar, Karen Harris-Shultz, Hongliang Wang, Marin Talbot Brewer, Leilani Sumabat, Pingsheng Ji

**Affiliations:** 1 Department of Plant Pathology, Coastal Plain Experiment Station, University of Georgia, Tifton, Georgia, United States of America; 2 United States Department of Agriculture-Agriculture Research Service (USDA-ARS), Crop Genetics and Breeding Research Unit, Tifton, Georgia, United States of America; 3 Department of Plant Pathology, University of Georgia, Athens, Georgia, United States of America; Tallinn University of Technology, ESTONIA

## Abstract

Fusarium wilt of watermelon, caused by *Fusarium oxysporum* f. sp. *niveum* (FON), occurs worldwide and is responsible for substantial yield losses in watermelon-producing areas of the southeastern United States. Management of this disease largely relies on the use of integrated pest management (i.e., fungicides, resistant cultivars, crop rotation, etc.). Knowledge about race structure and genetic diversity of FON in the southeastern US is limited. To determine genetic diversity of the pathogen, FON isolates were collected from symptomatic watermelon plants in commercial fields in Georgia and Florida, USA, and identified based on morphological characteristics and PCR analysis using FON-specific primers. Discriminant analysis of principal components (DAPC) of 99 isolates genotyped with 15 simple sequence repeat (SSR) markers grouped the isolates in eight distinct clusters with two prominent clusters (clusters 1 and 8). Cluster 1 consisted of a total of 14 isolates, out of which 85.7% of the isolates were collected in Florida. However, most of the isolates (92.4%) in cluster 8 were collected in Georgia. Both DAPC and pairwise population differentiation analysis (Ф_PT_) revealed that the genetic groups were closely associated with geographical locations of pathogen collection. Three races of FON (races 0, 2 and 3) were identified in the phenotypic analysis; with race 3 identified for the first time in Georgia. Overall, 5.1%, 38.9% and 55.9% of the isolates were identified as race 0, race 2 and race 3, respectively. The majority of the isolates in cluster 1 and cluster 8 belonged to either race 2 (35.6%) or race 3 (45.8%). Additionally, no relationship between genetic cluster assignment and races of the isolates was observed. The information obtained on genotypic and phenotypic diversity of FON in the southeastern US will help in development of effective disease management programs to combat Fusarium wilt.

## Introduction

Watermelon (*Citrullus lanatus*) is an economically important crop belonging to the Cucurbitaceae family, with a farm gate value of $124 million in GA [1]. Watermelon production can be severely affected by a vascular disease, Fusarium wilt [2, 3, 4, 5, 6]. Fusarium wilt of watermelon is caused by a soilborne fungal pathogen, *Fusarium oxysporum* f. sp. *niveum* (FON), which is host specific to watermelon. FON was first identified by E. F. Smith in South Carolina and Georgia [7]. Four races of FON (0, 1, 2 and 3) have been identified based on their aggressiveness to overcome specific resistance in a set of differential cultivars; Sugar Baby, Charleston Gray, Calhoun Gray and PI-296341-FR [8, 9, 10, 11, 12].

FON race 0 was first reported in Florida in 1963, and this race is not of high economic importance as most of the commercial watermelon cultivars possess resistance to the race 0 *Fo-1* gene to the pathogen [13]. FON race 1 is widespread throughout the watermelon-producing areas in the US, and many diploid (seeded) and a few newly released triploid (seedless) varieties possess resistance towards race 0 and 1 [3, 9]. FON race 2 was first reported from Israel (1976) and later reported in the US (1981) [13]. It is aggressive on both seeded as well as seedless watermelon cultivars and a high level of race 2 resistance is not available in commercial watermelon varieties making it an economically important issue for the watermelon growers in the US. Contrastingly, the watermelon pollinizer varieties developed by Syngenta Seeds, Inc., Super Pollinizer 5 and 6, have shown some promise as race 2 wilt resistance differentials. The Fusarium wilt resistance in these pollinizers was derived from PI-296341-FR [14]. Race 3 of FON was first identified in Maryland and later in Florida and is more aggressive than the other three races (0, 1 and 2) [6, 15].

Management of Fusarium wilt is challenging both due to long-term survival of chlamydospores in the soil and the evolution of new races [10]. Current management options for this disease include the use of seed treatments, crop rotation, soil fumigation, and chemical management, but in most cases, they are inadequate to manage FON epidemics [16]. In terms of chemical management, only prothioconazole (Proline, Bayer Crop Science) is registered for use against this pathogen [17]. Limited benefits can be observed in fields with low levels of infestation; however, in heavily infested fields, current chemical management practices are not effective. The phasing out of methyl-bromide has left even fewer options for managing this disease [18]. Because of the destructive nature of this disease, watermelon growers in the southeastern US often prefer resistant cultivars as the primary management option for Fusarium wilt [12, 19]. However, resistant commercial triploid cultivars are available only against races 0 and 1, and widespread distribution of FON race 2 renders this strategy ineffective [3, 20].

It has long been known that FON isolates from watermelon are highly diversified with different races, but little is known about the genetic diversity of FON and the potential relationship between genetic diversity and other traits, such as race, or geographical location. Furthermore, no recent studies have been carried out to examine the genetic diversity of FON in the US. In addition, phenotypic diversity of FON populations in the southeastern US has not been reported, yet this information is critical in devising effective management strategies to combat Fusarium wilt of watermelon. Thus, the objectives of this study were i) to examine the genetic and phenotypic (race) diversity of FON isolates collected from watermelon in the southeastern US, and ii) to determine if FON isolates are genetically structured by race or geographic location.

## Materials and methods

### *Fusarium oxysporum* f. sp. *niveum* isolation

FON isolates were collected from different watermelon-producing counties in GA and FL in 2012 and 2013 (Table 1). Isolates from Tift County, GA were collected in 2012 and isolates from Berrien County, GA; Cook County, GA and FL were collected in 2013. Isolates from the same field and county location were assigned to the same population. For example, all the isolates from Berrien County, GA were assigned as population 1 and all the isolates from Cook County, GA were assigned as population 2. Overall a total of four populations were assigned to all the isolates collected from GA and FL. The sampling fields were located more than 15 km apart. In each field, diagonal sampling method was used to take diseased plant samples with sampled plants located more than 10 meters apart. FON was isolated from symptomatic watermelon plant stem sections, by surface disinfecting the hypocotyl region with 0.6% sodium hypochlorite for 2 min, rinsing with sterile distilled water, air dried and placed on selective peptone pentocholoronitrobenzene agar (PPA) plates. After seven days of incubation in darkness at 25°C, FON colonies were then identified based on spore morphology [21] and using FON-specific primers Fn-1/Fn-2 [22] and Fon-1/Fon-2 [23]. Putative FON isolates were grown on potato dextrose agar (PDA) plates by incubating the plates at 25°C for seven days prior to DNA extraction. Briefly, total fungal genomic DNA was extracted using DNeasy Plant Mini kit (Qiagen, Valencia, CA) and 10 ng of DNA was amplified. The amplification was performed in a MyCycler thermal cycler (Bio-Rad Laboratories, Hercules, CA) using the protocol described by Lin et al. [23]. Upon confirming the isolates to be FON, single spore isolates were prepared as described previously [21]. These single spore FON isolates were further used in genetic and phenotypic diversity studies.

**Table 1:** Location of isolation, genetic cluster assignment, and races of *Fusarium oxysporum* f. sp. *niveum* isolates from watermelon in Georgia and Florida.

Isolate	Location	Genetic cluster^1^	Disease incidence (%)^2^				Race Identified^3^
			Sugar Baby	Charleston Gray	Calhoun Gray	PI-296341-FR	
F1-2^a^	Berrien, GA	8	100	100	100	90	3
F1-4 ^a^	Berrien, GA	8	100	100	70	0	2
F1-5a ^a^	Berrien, GA	8	100	100	100	70	3
F1-5b ^a^	Berrien, GA	8	100	100	100	70	3
F1-7 ^a^	Berrien, GA	8	100	100	70	0	2
F1-8 ^a^	Berrien, GA	8	100	100	100	80	3
F1-9 ^a^	Berrien, GA	8	100	100	100	80	3
F1-12 ^a^	Berrien, GA	8	100	100	90	0	2
F1-13 ^a^	Berrien, GA	8	100	90	90	0	2
F1-14 ^a^	Berrien, GA	8	100	100	90	0	2
F1-15 ^a^	Berrien, GA	8	100	100	90	0	2
F1-16 ^a^	Berrien, GA	8	95	5	0	0	0
F1-17 ^a^	Berrien, GA	8	100	100	70	0	2
F1-18 ^a^	Berrien, GA	8	100	100	100	90	3
F1-19 ^a^	Berrien, GA	8	100	100	100	80	3
F1-20 ^a^	Berrien, GA	8	100	100	80	0	2
F2-3 ^b^	Berrien, GA	8	100	100	90	0	2
F2-6 ^b^	Berrien, GA	8	100	100	80	0	2
F2-10 ^b^	Berrien, GA	8	100	100	90	0	2
F2-28 ^b^	Berrien, GA	8	100	100	100	70	3
F2-34 ^b^	Berrien, GA	8	95	100	90	85	3
F2-39 ^b^	Berrien, GA	8	100	100	100	70	3
F3-1 ^c^	Cook, GA	8	100	100	80	0	2
F3-2 ^c^	Cook, GA	8	100	100	100	70	3
F4-3 ^d^	Cook, GA	8	100	100	100	90	3
F3-7 ^c^	Cook, GA	8	100	100	100	80	3
F3-20 ^c^	Cook, GA	8	100	100	100	80	3
F3-22 ^c^	Cook, GA	8	100	100	100	90	3
F3-23 ^c^	Cook, GA	8	100	100	100	80	3
F3-24 ^c^	Cook, GA	8	100	100	100	80	3
F3-26 ^c^	Cook, GA	8	100	100	100	90	3
F3-29 ^c^	Cook, GA	8	95	100	80	85	3
F3-34 ^c^	Cook, GA	8	100	100	100	80	3
122e ^e^	Tift, GA	8	100	100	90	0	2
122g ^e^	Tift, GA	8	90	85	90	10	2
12105b ^e^	Tift, GA	8	85	85	90	0	2
13–101	FL	8	100	100	100	90	3
13–119	FL	8	100	100	90	95	3
13–134	FL	8	100	100	90	75	3
13–124	FL	7	95	85	85	90	3
12065 ^e^	Tift, GA	6	90	95	95	10	2
122a ^e^	Tift, GA	5	90	85	80	0	2
12126b ^e^	Tift, GA	4	80	80	80	80	3
F2-18 ^b^	Berrien, GA	3	100	0	0	0	0
13–115	FL	2	90	80	100	70	3
123a ^e^	Tift, GA	1	90	0	0	0	0
12105a ^e^	Tift, GA	1	90	90	90	70	3
13–102	FL	1	85	80	95	85	3
13–111	FL	1	85	90	85	80	3
13–112	FL	1	100	100	80	0	2
13–113	FL	1	90	85	90	90	3
13–114	FL	1	100	90	90	90	3
13–117	FL	1	100	100	80	0	2
13–118	FL	1	100	100	90	0	2
13–132	FL	1	90	90	80	85	3
13–133	FL	1	90	80	80	10	2
13–135	FL	1	90	90	100	80	3
13–136	FL	1	100	100	70	0	2
13–159	FL	1	95	100	85	5	2
*Race 0	GA		95	0	0	0	0
*Race1	GA		90	85	0	0	1
*Race 2	SC		95	90	90	0	2

^1^Genetic cluster assignment was determined by the use of simple sequence repeat markers.

^2^Mean disease incidence was recorded as the number of seedlings showing Fusarium wilt symptoms to the total number of seedlings planted (*n* = 10 seedlings/isolate/cultivar/experiment).

^3^Seedlings with ≥33% wilt incidence was considered as susceptible whereas seedlings with < 33% wilt incidence was considered as resistant (Martyn and Bruton, 1989).

^a-e^Isolates with same letters indicate isolates collected from same field.

Berrien, GA; Cook, GA and FL isolates were collected in 2013; Tift, GA isolates were collected in 2012.

*Reference isolates used for race 0, race 1 and race 2.

### Determining genetic diversity of *Fusarium oxysporum* f. sp. *niveum* isolates

Ninety-nine single spore FON isolates were genotyped using 15 published SSR markers for *F*. *oxysporum* [24, 25] (Table 2). Fungal DNA was extracted as mentioned above and a NanoDrop spectrophotometer (Thermo Fisher Scientific, Waltham, MA) was used to quantify the DNA concentrations. To determine the best annealing temperature, a gradient PCR was performed at annealing temperatures ranging from 50 to 60°C using the 15 SSR primer pairs and two isolates (F1-4, F4-3). An annealing temperature of 55°C generated the best polymorphic profile and was then used for subsequent studies. PCR was performed in a 10 μl of reaction mixture containing 2 μl of 5x Clear GoTaq Flexi Buffer (Promega Corp., Madison, WI), 1 μl of 25 mM MgCl_2_, 0.8 μl of 2.5 mM dNTP, 0.5 μl of 1 μM M13 tagged forward primer, 2 μl of 1 μM reverse primer, 1.8 μl of 1 μM of M13 primer (M13-TGTAAAACGACGGCCAGT) fluorescently labeled with IRD Dye 700 CW fluorophore (Eurofins MWG Operon, Huntsville, AL), 0.04 μl of GoTaq Flexi DNA polymerase, 0.86 μl of PCR grade water (Teknova, Hollister, CA) and 1 μl of FON DNA (2.5 ng/μl). The amplification was performed in a T100 thermal cycler (Bio-Rad Laboratories, Hercules, CA) using a protocol as described by Li et al. [26]. Briefly, the PCR conditions included: initial denaturing at 94°C for 3 min, followed by 39 cycles of denaturing at 94°C for 30 s, annealing at 55°C for 1 min, and polymerization at 72°C for 70 s, with a final extension step at 72°C for 10 min. The PCR product (2 μl) was combined with 5 μl of Blue Stop (LI-COR Biosciences, Lincoln, NE) and 0.35 μl of this mixture was loaded on an acrylamide gel (6.5%) using a LI-COR Biosciences 4300 DNA analyzer. A 50–700 bp ladder was used and alleles for each locus were scored based on DNA fragment size.

**Table 2:** Primer sequences, SSR motifs and allele sizes of 99 *Fusarium oxysporum* f. sp. *niveum* isolates.

Primer	Primer sequence (5’–3’)	*T*_a_*	SSR motif	Allele size
MB2	F: TGCTGTGTATGGATGGATGG R: CATGGTCGATAGCTTGTCTCAG	57	(GT)11(GA)6	234, 237, 238, 240, 242, 246, 248, 250, 252, 254, 260, 264, 271, 275
MB5	F: ACTTGGAGGAAATGGGCTTC R: GGATGGCGTTTAATAAATCTGG	54	(TG)9	252, 254, 256, 267, 274, 344
MB9	F: TGGCTGGGATACTGTGTAATTG R: TTAGCTTCAGAGCCCTTTGG	51	(CA)9	126, 105, 130, 141, 234, 237, 240, 254
MB10	F: TATCGAGTCCGGCTTCCAGAAC R: TTGCAATTACCTCCGATACCAC	48	(AAC)6	206, 208
MB11	F: GTGGACGAACACCTGCATC R: AGATCCTCCACCTCCACCTC	68	(GGC)7	172, 175, 177, 180, 182, 186
MB13	F: GGAGGATGAGCTCGATGAAG R: CTAAGCCTGCTACACCCTCG	68	(CTTGGAAGTGGTAGCGG)14	144, 264, 296, 376, 382, 345, 395, 400, 422, 476, 483, 492, 500
MB14	F: CGTCTCTGAACCACCTTCATC R: TTCCTCCGTCCATCCTGAC	57	(CCA)5	183, 184,186
MB17	F: ACTGATTCACCGATCCTTGG R: GCTGGCCTGACTTGTTATCG	57	(CA)21	299, 301, 303, 308, 312, 317, 319, 320, 321, 331, 334, 337, 339
MB18	F: GGTAGGAAATGACGAAGCTGAC R: TGAGCACTCTAGCACTCCAAAC	57	(CAACA)6	284, 289, 293
Fo9	F: GGCAGAAAAGATACTGAACG R: TTGAATTGCCAACTCTTCTT	55	(GGA)11	204
Fo120	F: GAAAGTGGATGGAAGAAAGA R: GATAGGCTGTTGTTGTGGTT	54.8	(CAG)7	300
Fo310	F: CATTGCAGCAGGAATTAGAT R: CTAGGTAGGCATACGAGGGT	55.6	(CA)7	318
Fo314	F: GAAAAGGAGAGACTGCAAAA R: CTCTTCTTCCTCGTGTTGAC	54.8	(GAA)4 (GAG)9 (GAG)4	297
Fo671	F: TGTTCCCTGAGTTGGTAGTC R: CTCCCAATCAGATCCTTCT	55.0	(CAG)8	111
Fo1513	F: TGTTCCCTGAGTTGGTAGTC R: CTCCCAATCAGATCCTTCT	55.0	(GGA)9	125

* PCR annealing temperature.

### Identification of clonal genotypes

Clones are genetically identical individuals that arise due to asexual reproduction. However, based on the sample size, the number of markers used, the number of alleles for each marker, and the allele frequencies, the same MLG may arise by recombination or outcrossing, and not necessarily be indicative of a clone. To confirm that the repeated genotypes are clones and not arising from an outcrossing event, we used MLGsim v2.0 [27] to calculate the probability (ρ_sex_) that each MLG could have occurred from an outcrossing event [28]. A significant ρ_sex_ value indicates that the repeated MLG likely arose from clonal reproduction. The P values for ρ_sex_ were estimated by 1000 random permutations of the data.

### Analyses of population structure

To determine the genetic diversity within and genetic structure among FON populations, GenAIEx v.6.5 [29] was used to estimate the number of alleles and unbiased gene diversity (*h*) for each SSR locus. Analysis of molecular variance (AMOVA) was used to calculate genetic differentiation (Ф_PT_) among populations. To estimate the major patterns of variation within and among populations from different geographical locations, a principal coordinate analysis (PCoA) was conducted with GenAIEx. AMOVA was also used to calculate genetic variation within and among different FON races. To determine if two individuals taken at random have unique genotypes, genotypic diversity (Ĝ) was estimated in the R package adegenet v 1.4–1 [30, 31]. In order to understand FON population structure without a priori assignment of individuals to populations, the number of clusters among FON sampled from different locations in southeastern US and the assignment of FON isolates to each cluster. Discriminant analysis of principal components (DAPC) was used by applying R package adegenet v 1.4–1 [30, 31]. DAPC clusters were determined by K-means clustering of principal components, where K was inferred as the number of cluster where the Bayesian information criteria (BIC) increases or decreases by a negligible amount.

### Phenotypic diversity (race typing)

Fifty-nine single spore FON isolates were selected for race typing. The fifty-nine single spore isolates selected for race typing were representative of each field and county location in GA and FL. The set of differential cultivars used to determine FON races under greenhouse conditions included: Sugar Baby (no resistance to any race), Charleston Gray (resistant to race 0), Calhoun Gray (resistant to race 1), and PI-296341-FR (resistant to race 2) [6]. Reference FON isolates for race 0, race 1 (race 0 and race 1 were received from Hunt Sanders, University of Georgia, Tifton, GA) and race 2 (received from Dr. Anthony Keinath, Clemson University, Charleston, SC) were included in each experiment as controls.

To prepare FON inoculum, isolates were grown on PDA at 25°C for 7 days. Five mycelial plugs (5 mm in diameter) from the edge of a growing colony were transferred aseptically to a 250-ml flask containing 200 ml quarter strength potato dextrose broth (PDB). The liquid culture was incubated for 14 days at 23±1°C on an orbital shaker (G10 Gyrotory Shaker, New Brunswick Scientific Company, NJ) at 150 rpm. Colonized liquid PDB was filtered through three layers of sterile cheesecloth and the concentration of spore suspension (>95% microconidia) was adjusted to 1×10^6^ spores/ml by adding sterile distilled water (SDW).

For greenhouse evaluation, watermelon seedlings of the four differentials were grown in pots (9-cm diameter) containing a mixture of sand:peat:vermiculite (4:1:1, v:v:v). Seedlings were inoculated at the first true-leaf stage by pipetting 5 ml of conidial suspension (1×10^6^ spores/ml) near the base of each watermelon seedling. Ten plants/isolate/cultivar in separate pots were inoculated and the same number of plants treated with SDW served as a negative control. After inoculation, seedlings were maintained at 28°C day and 20°C evening with 70–80% relative humidity in the greenhouse. The percentage of seedlings showing the characteristic symptoms of Fusarium wilt such as yellowing, stunting, or wilting were evaluated weekly and disease incidence was recorded after 4 weeks. Additionally, plants were also rated for disease severity at the end of 4 weeks using a scale from 0 to 9 with a score of 0 representing asymptomatic plants, 3 for plants with cotyledon lesions, 5 for plants showing slight wilting and stunting, 7 for plants with severe wilting and stunting and 9 for dead plants. Plants rated as 0 were classified as resistant, 1 or 3 as intermediate resistant and 5, 7 or 9 as susceptible [32]. For assessing disease reactions on each differential and isolate treatment, differential with ≥33% wilt incidence was considered as susceptible whereas differential with <33% wilt incidence was considered as resistant [33]. A total of two independent greenhouse experiments were conducted.

In the greenhouse studies, three symptomatic seedlings/isolate/differential were used to confirm if the symptoms observed were due to FON infection using the method previously described [17]. Briefly, seedlings were surface sterilized and placed on PPA plates [21]. The plates were incubated for two weeks at 25°C and checked periodically for putative FON colonies. Later, colonies were purified by sub-culturing hyphal tips on PPA plates and then transferring onto potato dextrose agar. The cultures were identified to be FON based on morphological characteristics [21] and PCR analysis using FON-specific primers Fn-1/Fn-2 [22] and Fon-1/Fon-2 [23] as described above.

## Results

### Genetic diversity of *Fusarium oxysporum* f. sp. *niveum* isolates

Of the 15 SSR loci used to genotype our 99 FON isolates, 5 were identified as monomorphic (MB9, MB10, MB13, MB18, and Fo310) and one (MB5) did not amplify for the majority of the isolates. Hence, FON population genetic analyses were based on 9 polymorphic SSR loci. The number of alleles for each of the 9 loci varied from 3 to 16. For the 99 FON isolates, 14 MLG were identified. It was observed that around 75% of the genotypes were repeated and hence the genotypic diversity for the 99 isolates was very low (Ĝ = 0.423). Additionally, for each population, the genotypic diversity was very low (Ĝ <0.423) except for FL population (Ĝ = 0.600) (Table 3). Rarefied allelic richness for all the populations ranged from 2.90 to 4.85 indicating that most loci for the populations showed over four alleles when standardized for sample size. Contrastingly, allelic evenness, which is the measure of the distribution of genotype abundances ranged from 0.391 to 0.706. All the GA populations had allelic evenness values of ≤0.50 and the FL population had an allelic evenness value of 0.706, indicating that all the MLG’s observed in the FL population are closer to equal abundance than those in the GA populations (Table 3). Higher genotypic diversity value (Ĝ = 0.600) with a higher allelic evenness value (0.706) for the FL population suggests that the FL population is more diverse than the GA populations.

**Table 3:** Estimates of genetic diversity for *Fusarium oxysporum* f. sp. *niveum* population from the southeastern United States.

Population^a^	No. of isolates (N)	No. of MLG (g)	Genotypic diversity (Ĝ)^b^	Allelic richness^c^	Allelic evenness^d^
GA1	40	5	0.188	2.90	0.391
GA2	19	3	0.194	3.00	0.475
GA3	20	5	0.420	4.85	0.500
FL	20	5	0.600	4.85	0.706
Total	99	14	0.423	4.25	0.400

^a^ Four populations were sampled: Berrien county, Georgia (GA1), Cook county, Georgia (GA2), Tift county, Georgia (GA3), Florida (FL).

^b^ Ĝ is the genotypic diversity that represents the probability of two randomly selected individuals having unique multilocus genotypes (MLG), and was calculated using R package adegenet v 1.4–1 (Jombart 2008; Jombart and Ahmed 2011).

^c^ Allelic richness measures the number of observed MLGs.

^d^ Allelic evenness measure of distribution of genotype abundances.

Six repeated MLG’s (g_2_) were found across the populations from the southeastern US (Table 4). The ρ_sex_ value associated with all the repeated MLG’s was highly significant (p<0.001) indicating that these repeated MLG’s did not likely arise from recombination, and were the result of clonal reproduction (Table 4). Moreover, within each sampled population there were many clonal MLG’s containing multiple members. For instance, GA1 contained a repeated MLG consisting of 36 isolates while GA2, GA3 and FL contained around 17 members, respectively. Also, MLG 11 was shared among all the four populations (Table 3, labeled as x_11_) and MLG 14 was shared among two populations (Table 4, labeled as y_14_).

**Table 4:** Clonal composition of *Fusarium oxysporum* f. sp. *niveum* populations from the southeastern United Sates.

Population^a^	N	g^b^	g_2_^c^	Number of isolates in each repeated MLG^d^
GA1	40	5	1	36^x11^*
GA2	19	3	1	17 ^x11^*
GA3	20	5	2	15 ^x11^*, 2 ^y14^*
FL	20	5	2	6 ^x11^*, 11 ^y14^*
Total	99	14	6	74 ^x11^*, 13 ^y14^*

^a^ Four populations were sampled: Berrien county, Georgia (GA1), Cook county, Georgia (GA2), different counties, Georgia (GA3), Florida (FL).

g^b^ Number of MLG in each population.

g_2_^c^ Number of MLG represented by more than one member (repeated MLG).

^d^ Superscripts of “x” or “y” indicate MLG that were present in more than one population, with “X” representing MLG present in three or more different populations and “y” indicating repeated MLG in two different populations.

* Significant (p < 0.001) ρ_sex_ estimate

Superscript number indicates the repeated MLG to which the isolates belong.

### Population structure of *Fusarium oxysporum* f. sp. *Niveum*

**Fig 1:**
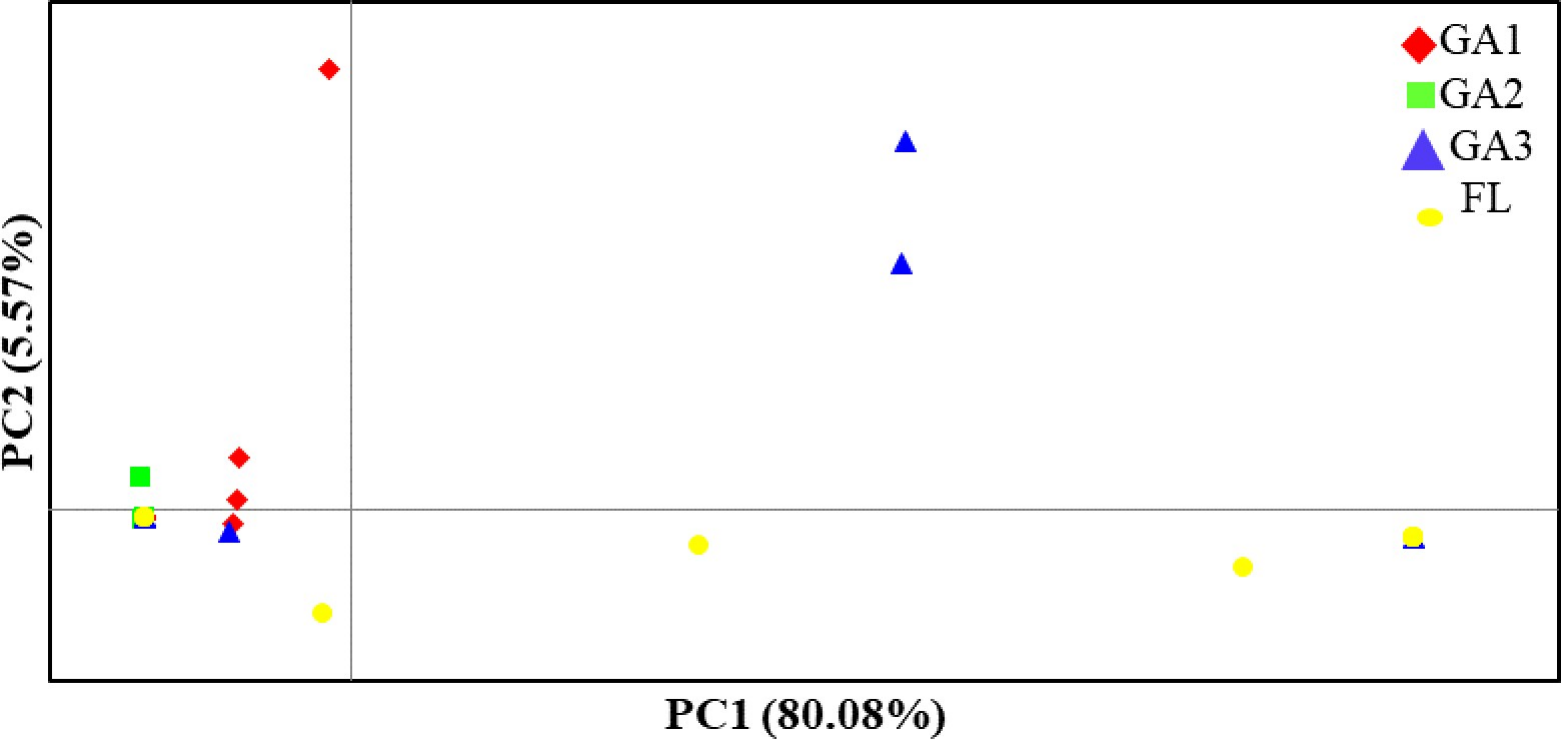
Principal coordinate analysis of four populations of *Fusarium oxysporum* f. sp. *niveum* isolates collected from watermelon fields in southeastern United States. Sampling locations include Berrien County, Georgia (GA1), Cook County, Georgia (GA2), Tift County, Georgia (GA3), and Florida (FL). PC1 (80.08%) and PC2 (5.57%) refer to the first and the second coordinate.

Pairwise population differentiation (Ф_PT_) was performed on the four populations. Significant differences were observed between the FL population and GA1, GA2, GA3 populations (P≤0.002) (Table 5), indicating genetic structure between populations. Among GA populations, significant differences were observed only among GA3 and GA1 populations (P≤0.01) (Table 5). For the PCoA analysis the first and second principal coordinates explained 80.0% and 5.6% of the variance, respectively (Fig 1). The GA isolates belonging to three distinct populations were clustered together and less diverse. The Florida population, on the other hand, was more diverse.

**Table 5:** Geographical population structure measured by Ф_PT_ between *Fusarium oxysporum* f. sp. *niveum* populations in the southeastern United States.

			Population^a^	
Population	GA1	GA2	GA3	FL
GA1	…			
GA2	0.00 ^ns^	…		
GA3	0.122**^b^	0.096 ^ns^	…	
FL	0.651***	0.585 ***	0.321***	…

^a^ Four populations were sampled: Berrien county, Georgia (GA1), Cook county, Georgia (GA2), Tift county, Georgia (GA3), Florida (FL).

^b^ Pairwise population differentiation was measured by Excoffier’s Ф_PT_ (Excoffier et al.1992), an analog of *F*_*st*_ analysis measured via analysis of molecular variance, using GenAlEx v.6.503 (Peakall and Smouse 2006).

Significance levels (ns = not significant, * = P≤0.05, ** = P≤0.01, *** = P≤0.002) are based on P values determined by 1000 permutations of the data.

Partitioning of the genetic variation (Ф_PT_) was performed for the three FON races detected (0, 2 and 3). Significant partitioning of the genetic variation by race was not observed (Table 6). Moreover, molecular variance among races accounted for 0% of the variation, while 100% of the variation was observed within the races indicating high diversity within each race (Table 7).

**Table 6:** Population structure measured by Ф_PT_ between *Fusarium oxysporum* f. sp. *niveum* races in the southeastern United States.

		Race^b^	
Race^a^	0	2	3
0	…		
2	0.129 ^ns^	…	
3	0.083^ns^	0.000 ^ns^	…

^a^ Three races of *Fusarium oxysporum* f. sp. *niveum* from GA and FL were identified.

^b^ Pairwise population differentiation was measured by Excoffier’s Ф_PT_ (Excoffier et al.1992), an analog of *F*_*st*_ analysis measured via analysis of molecular variance, using GenAlEx v.6.503 (Peakall and Smouse 2006).

Significance levels (ns = not significant, * = P≤0.05, ** = P≤0.01, *** = P≤0.002) are based on P values determined by 1000 permutations of the data.

**Table 7:** Analysis of molecular variance for three *Fusarium oxysporum* f. sp. *niveum* races collected from four different locations in the southeastern United States.

Source of variation	d.f.	S.S	M.S.	Variance%
Among races	2	3.167	1.583	0%
Within races	53	81.869	1.545	100%
Total	55	85.036	3.128	

d.f., Degree of freedom; S.S, Sum of squared differences; M.S., Mean square

**Fig 2:**
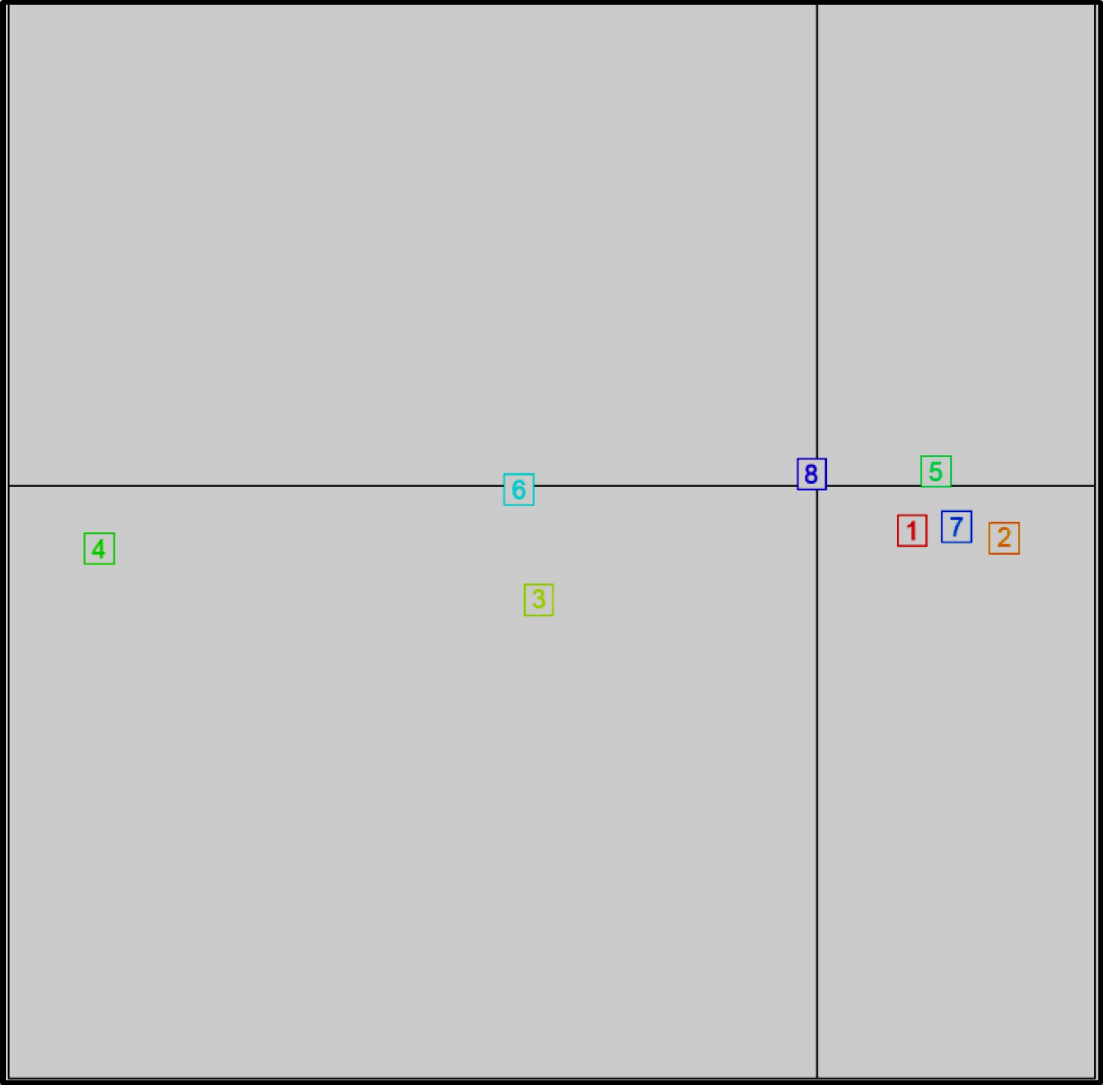
Discriminant analysis of principal components for *Fusarium oxysporum* f. sp. *niveum* (FON) from the southeastern United States showing scatterplot of the 8 assigned clusters based on Bayesian information criterion. The four populations include: Berrien County, Georgia (GA1), Cook County, Georgia (GA2), Tift County, Georgia (GA3), and Florida (FL).

**Fig 3:**
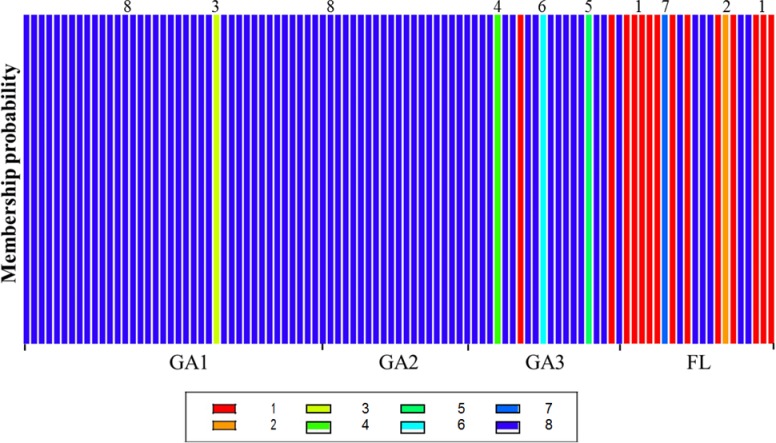
Discriminant analysis of principal components for *Fusarium oxysporum* f. sp. *niveum* (FON) from the southeastern United States showing a histogram of assignment probability of 99 FON isolates from 4 field populations into eight genetic clusters. The four populations include: Berrien County, Georgia (GA1), Cook County, Georgia (GA2), Tift County, Georgia (GA3), and Florida (FL).

DAPC supported K = 8 clusters. None of the clusters in DAPC overlapped (Fig 2). Clusters 2 through 7 each represented a single FON isolate whereas cluster 1 and 8 represented 14 and 79 FON isolates, respectively (Fig 3). Additionally, we identified some clusters which represented isolates that belong to a dominant or prevalent clone, clones with genotypes containing similar alleles and unique genotypes within a dominant clone. It was observed that some clusters were distributed across multiple populations. For instance, cluster 1 contained one of the dominant clones (14 FON isolates) detected mostly in the FL population. On the other hand, most of the GA isolates belonged to cluster 8 (represented by navy blue) containing 79 members from the three GA locations (Fig 3). The other clusters represented different MLG’s containing very few members.

### Phenotypic diversity

Fifty-nine FON isolates from the eight genetic clusters identified above (K = 8) (Fig 3) were selected for race determination. Overall, 5.1% (3/59), 38.9 (23/59), and 55.9% (33/59) of the isolates were identified as race 0, race 2 and race 3, respectively. The majority of the isolates used for race identification were from cluster 1 (14/59) and cluster 8 (39/59). Among cluster 1, the majority of the isolates were collected from FL (85.7%) (12/14) and likewise a majority of cluster 8 isolates were collected from GA (36/39). Among the isolates in cluster 1, 7.1% (1/14), 42.8% (6/14) and 50% (7/14) were identified as race 0, race 2 and race 3, respectively (Table 1). Among the isolates in cluster 8, 2.6% (1/39), 38.5% (15/39) and 58.9% (23/39) were identified as race 0, race 2 and race 3, respectively. None of the isolates in clusters 1 or 8 were identified as race 1.

With respect to geographical location, GA isolates accounted for 14.3% (2/14) of cluster 1, out of which 7.1% (1/14) each were identified as race 0 and race 3, respectively. In contrast, FL isolates from the same cluster accounted for 85.7% (12/14), and 42.8% (6/14) of the isolates were identified as race 2 and race 3, respectively. Cluster 8 includes 66.1% (39/59) of the race identified isolates, out of which 61% (36/59) were collected in GA, where 2.6% (1/39), 38.5% (15/39) and 51.3% (20/39) identified as race 0, 2 and 3, respectively. Florida isolates in the same cluster accounted for only 7.7% (3/39) of the race identified isolates, all of which belonged to race 3 only. Other clusters (2, 3, 4, 5, 6 and 7) included only one isolate each either identified as race 0, race 2 or race 3 (Table 1). For instance, cluster 3, 4, 5 and 6 contained one isolate each from GA identified as race 0, race 3 and race 2, respectively. However, cluster 2 and cluster 7 contained one isolate each from FL identified as race 3, respectively (Table 1). Disease severity of watermelon differentials inoculated with FON isolates belonging to either race 2 or race 3 ranged from 5 to 9, respectively. Seedlings treated with SDW remained asymptomatic. One hundred percent of the seedlings assayed for putative FON symptoms in the greenhouse studies were confirmed as FON by specific PCR assays as described above.

## Discussion

The population genetic structure of FON in the southeastern US was investigated based on nine published SSR markers [24, 25]. In the current study, we identified eight genetic clusters within FON populations from watermelon fields in GA and FL with the majority of isolates belonging to two of the clusters (1 and 8) (Fig 3). Cluster 1 consists of isolates collected from FL (85.7%) whereas most of the isolates (92.4%) in cluster 8 were collected from GA. This suggests that the populations from GA and FL are different; however, some isolates from GA clustered with FL isolates and vice versa. This could be from movement of FON between the two states through infested seeds or watermelon transplants. Hence, further population studies of FON isolates from seed and/or transplants may shed light on this observation.

Among the FON populations collected throughout the southeastern US, we observed clones that occurred at a high frequency and were widespread at the regional level. Overall, six clones were distributed across the four populations obtained from GA and FL. Additionally, most of the isolates were associated with only few MLG’s (11 and 14). One clone was distributed across all four populations (GA1-GA3 and FL), whereas another clone was only distributed across two populations (GA3 and FL). Among the four FON populations, clones represented 87 of the 99 FON isolates (Table 4). Similar observations of clonal populations with limited genotypic diversity were reported in other *Fusarium oxysporum* pathosystems. For example, in *Fusarium oxysporum* f. sp. *cubense* (causal agent of Fusarium wilt of banana), fungal populations from different geographical locations were clonal. Ten clonal lineages were identified, and among them, the two largest lineages had pantropical distribution [34]. Similarly, *Fusarium oxysporum* f. sp. *albedinis* (causal agent of Fusarium wilt of date palm) isolates collected from different geographical locations in Morocco were found to be less diverse and clonal [35].

The diversity among GA populations was comparatively lower (genotypic diversity≤0.420, allelic evenness≤0.50); whereas, the FL population had a higher diversity than the GA populations (genotypic diversity = 0.600, allelic evenness = 0.706) (Table 3). The reason behind the prevalence and extensive presence of clones is unclear. However, one possible reason could be related to the presence of asexual propagules like chlamydospores, which are reported to survive in the soil for up to 16 years [10]. These propagules occupy small, discrete territories and can be spatially rearranged when the soil is cultivated or irrigated. The propagules may propagate clonal lineages for a brief period of time or for many seasons either locally or over a wide geographical area [36]. These asexually produced chlamydospores can disperse clonal lineages through movement of infested seeds, transplants, irrigation water and agricultural machinery. Prevalent and widespread clones were also reported in case of *Phytophthora infestans* [37] and *Sclerotinia sclerotiorum* [36] and *Stagonosporopsis citrulli* [38].

The genetic structure of the FON populations was identified at the regional level based on both Ф_PT_ statistics and analysis of variance of principal components. Significant differences were observed between GA and FL populations (Table 5). Also, among GA population differences were observed only between GA3 and GA1 populations (Table 5). This indicates that gene flow is more prevalent among fields in GA than between fields in GA and FL. Additionally, DAPC revealed that the populations in GA and FL were distinct. The majority of isolates in cluster 8 were collected from GA (92.4%) whereas majority of isolates in cluster 1 were collected from FL (85.7%).

Phenotypic diversity of representative isolates from eight clusters revealed that race 2 and race 3 are the predominant races in both states. More than 90% of the FON isolates from the 8 clusters belonged to race 2 or race 3. Less than 5% of the isolates belonged to race 0. This is the third instance where FON race 3 was identified in the southeastern US. Recently, FON race 3 was identified in three different counties of Florida (Madison, Levy, and Lee) [15]. Prior to FL, race 3 was identified in Maryland [6]. In GA, FON race 2 was identified by Bruton et al. in 2008 [39]; however, race 3 had never been reported in GA prior to this study. The detection of race 3 in GA could be due to introduction and establishment of the pathogen through contaminated seeds or transplants harboring FON race 3. Race typing of FON isolates from contaminated seed sources may shed light on this hypothesis. Alternatively, it could be hypothesized that extensive use of watermelon with race 2-resistant pollinizers in infested soil could pose high selection pressure for the evolution of race 3 isolates.

The phenotypic study revealed that few isolates were race 0. In addition, none of the isolates were identified as race 1. It is plausible that race 0 and 1 may be an ancestral race and that races 2 and 3 may be of more recent origin [13]. Additionally, when molecular variance among different races was calculated, 100% of the variation was observed within each race rather than among different races. It is possible that isolates from different races are mutants of the same background genotype and the most virulent mutants predominate as observed by Nui et al. [40]. The researchers observed that an effector gene, *FONSIX6* is present in race 0 and race 1 but absent in race 2. Further they speculated that race 0 can be a less aggressive form of race 1. In *Fusarium oxysporum* f. sp. *lycopersici*, *SIX* genes (avirulence genes) are located in chromosome 14 (supernumerary chromosome or pathogenicity chromosome) and are responsible for virulence/aggressiveness in different races of the pathogen. The researchers claim that evolution of new races can be attributed to the horizontal transfer of this supernumerary chromosome. Although, *FONSIX6* was determined in FON race 0 and race 1, its location in the chromosome is still undetermined. If *FONSIX6* is located on a supernumerary chromosome, FON race 1 could have acquired this avirulence gene during by horizontal gene transfer, but this needs to be confirmed. Effector genes specific in FON race 2 and race 3, if any, are yet to be discovered. Further, it will be interesting to investigate the effector repertoire and their chromosome locations to shed some light in the evolution of FON race 2 and race 3.

Comparable results were observed in a previous study by Bruton et al. [39], where a lower percentage of isolates in GA were identified as race 2, e.g., out of eight isolates tested two belonged to race 2. It may be possible that race 0 is serving as the progenitor population for the evolution of other FON races due to widespread use of race 0 or 1 resistant watermelon cultivars. FON race 0 was first reported in Florida in 1963 [13] whereas FON race 2 was not reported until 1985 in Texas [41, 42]. We propose that with time, the populations of race 0 will continue to decline whereas those of race 2 and race 3 will continue to rise. This can be illustrated from the distribution frequencies of the different races in Georgia (Table 1) where out of the 59 isolates tested only 5% of the isolates belonged to race 0, and higher percentages of the isolates belonged to race 2 (38.9%) or race 3 (55.9%). Related results were also observed by Zhou and Everts [43] who reported more isolates of FON race 2 as compared to race 1 or 0 from a field in Maryland.

In our study, a strong correlation of FON genotypes with geographical locations as compared to pathological races was observed. Previous studies had similar observations where FON races did not correlate with the genetic diversity. Kim et al. [44] studied the genetic variation of 50 FON isolates encompassing three known races (0, 1 and 2) collected from different geographical locations using restriction fragment length polymorphisms (RFLP) and observed that there was no relationship between RFLP haplotypes, races or geographical locations. Additionally, they also observed that all the races were closely related and shared common sequences of chromosomal DNA [45].

In other *Fusarium oxysporum* pathosystems, similar observations were made. Castano et al. [46] studied the genetic and phenotypic diversity of *Fusarium oxysporum* f. sp. *dianthi* in southern Spain using random amplified polymorphic DNA (RAPD)-PCR, DNA sequence analysis of the *TEF1-α* gene, and race-specific molecular markers. High genetic homogeneity within the races in the investigated population was observed, which was consistent with earlier reported studies. Additionally, Alves-Santos et al. [47] studied 128 *F*. *oxysporum* f. sp. *phaseoli* strains in Spain using intergenic spacer (IGS) region polymorphism of ribosomal DNA, electrophoretic karyotype patterns, vegetative compatibility and pathogenicity analyses. No correlation between pathogenicity and VCG, IGS restriction fragment length polymorphism, or electrophoretic karyotype was observed. Contrastingly, in a study of *Fusarium oxysporum* f. sp. *vasinfectum* (FOV) (causal agent of Fusarium wilt of cotton), random amplified polymorphic DNA (RAPD) markers clustered the 46 FOV isolates into three groups based on geographical locations and pathological reactions. Three distinct virulence groups were identified that corresponded with three races 3, 4 and A [48].

In summary, this study provides new insights into the current status of genetic and phenotypic diversity of FON in the southeastern US. A new race of FON, race 3, was identified for the first time in GA and the population genetic analyses based on SSR markers was useful in describing the genetic diversity of FON isolates. The population genetic analyses divided the FON isolates from GA and FL into eight distinct clusters with two prominent clusters (cluster 1 mostly from FL and cluster 8 mostly from GA). Determining races and genetic diversity of FON prevalent in watermelon production in this region facilitates development of more effective disease management programs.

## Supporting information

S1 File MLGsim data for FON isolates used in the study. (CSV)

S2 File Principal coordinate analysis data for FON isolates used in the study. (XLSX)
